# Integrated image and location analysis for wound classification: a deep learning approach

**DOI:** 10.1038/s41598-024-56626-w

**Published:** 2024-03-25

**Authors:** Yash Patel, Tirth Shah, Mrinal Kanti Dhar, Taiyu Zhang, Jeffrey Niezgoda, Sandeep Gopalakrishnan, Zeyun Yu

**Affiliations:** 1 Department of Computer Science, University of Wisconsin-Milwaukee, Milwaukee, WI USA; 2 Advancing the Zenith of Healthcare (AZH) Wound and Vascular Center, Milwaukee, WI USA; 3 College of Nursing, University of Wisconsin Milwaukee, Milwaukee, WI USA; 4 Department of Biomedical Engineering, University of Wisconsin-Milwaukee, Milwaukee, WI USA

**Keywords:** Multi-modal wound image classification, Wound location Information, Body map, Combined image-location analysis, Deep learning, Convolutional neural networks, Transfer learning, Data mining, Machine learning, Image processing, Medical research, Health care

## Abstract

The global burden of acute and chronic wounds presents a compelling case for enhancing wound classification methods, a vital step in diagnosing and determining optimal treatments. Recognizing this need, we introduce an innovative multi-modal network based on a deep convolutional neural network for categorizing wounds into four categories: diabetic, pressure, surgical, and venous ulcers. Our multi-modal network uses wound images and their corresponding body locations for more precise classification. A unique aspect of our methodology is incorporating a body map system that facilitates accurate wound location tagging, improving upon traditional wound image classification techniques. A distinctive feature of our approach is the integration of models such as VGG16, ResNet152, and EfficientNet within a novel architecture. This architecture includes elements like spatial and channel-wise Squeeze-and-Excitation modules, Axial Attention, and an Adaptive Gated Multi-Layer Perceptron, providing a robust foundation for classification. Our multi-modal network was trained and evaluated on two distinct datasets comprising relevant images and corresponding location information. Notably, our proposed network outperformed traditional methods, reaching an accuracy range of 74.79–100% for Region of Interest (ROI) without location classifications, 73.98–100% for ROI with location classifications, and 78.10–100% for whole image classifications. This marks a significant enhancement over previously reported performance metrics in the literature. Our results indicate the potential of our multi-modal network as an effective decision-support tool for wound image classification, paving the way for its application in various clinical contexts.

## Introduction

Wound diagnosis and treatment are a pressing issue worldwide, with a considerable population suffering from wounds. As per a 2018 retrospective analysis, the costs for wound treatment have been estimated to be between $28.1 billion to $96.8 billion^1–3^, reflecting the tremendous financial and medical burden. The most commonly observed wounds include diabetic foot ulcer (DFU), venous leg ulcer (VLU), pressure ulcer (PU), and surgical wound (SW), each associated with a significant portion of the population^4–7^. Given these circumstances, effective wound classification is crucial for timely and adequate treatment.

Until recently, wounds were predominantly classified manually by specialists, often leading to inconsistencies due to lack of specific guidelines. However, the advent of artificial intelligence (AI) has brought about significant changes in healthcare, including wound diagnosis^8–14^. A cornerstone of this data-driven shift is the emergence of Deep Learning (DL), known for its prowess in autonomously analyzing complex data to unveil essential information, relationships, and patterns^9,15^. The landscape of DL is vast, encompassing various methodologies such as Convolutional Neural Networks (CNN), Deep Belief Networks (DBN), Deep Boltzmann Machines (DBM), Stacked Autoencoders, and many more. These techniques have been pivotal in advancing medical diagnostic fields, notably wound image analysis^16,17^. Among these, Deep Convolutional Neural Networks (DCNNs) stand out due to their multi-layered structure, which is a marked evolution from their predecessor models with fewer layers^18^. The principal mathematical operation in these networks is convolution, crucial for processing the input data^19^. The discipline of wound care has witnessed substantial strides through the adoption of DL^20–22^ particularly DCNNs, in wound image analysis tasks like segmentation^23,24^ and classification^25–27^. Numerous studies have accentuated the efficacy and efficiency of deep convolutional neural networks in advancing wound diagnosis and analysis^18–22^.

Notwithstanding the advancements, the accuracy of wound classification models remains constrained due to the partial information incorporated in the classifiers. The present research introduces an innovative approach to address this limitation by including wound location as a significant feature in the wound classification process. Wound location, a standard entry in electronic health record (EHR) documents, is instrumental in wound diagnosis and prognosis. A body map has been utilized to facilitate accurate and consistent wound location documentation^28^, enhancing the classifier’s performance by providing a more holistic set of data for classification. The classifier trained on both image and location features outperforms those reliant solely on image data.

**Figure 1:**
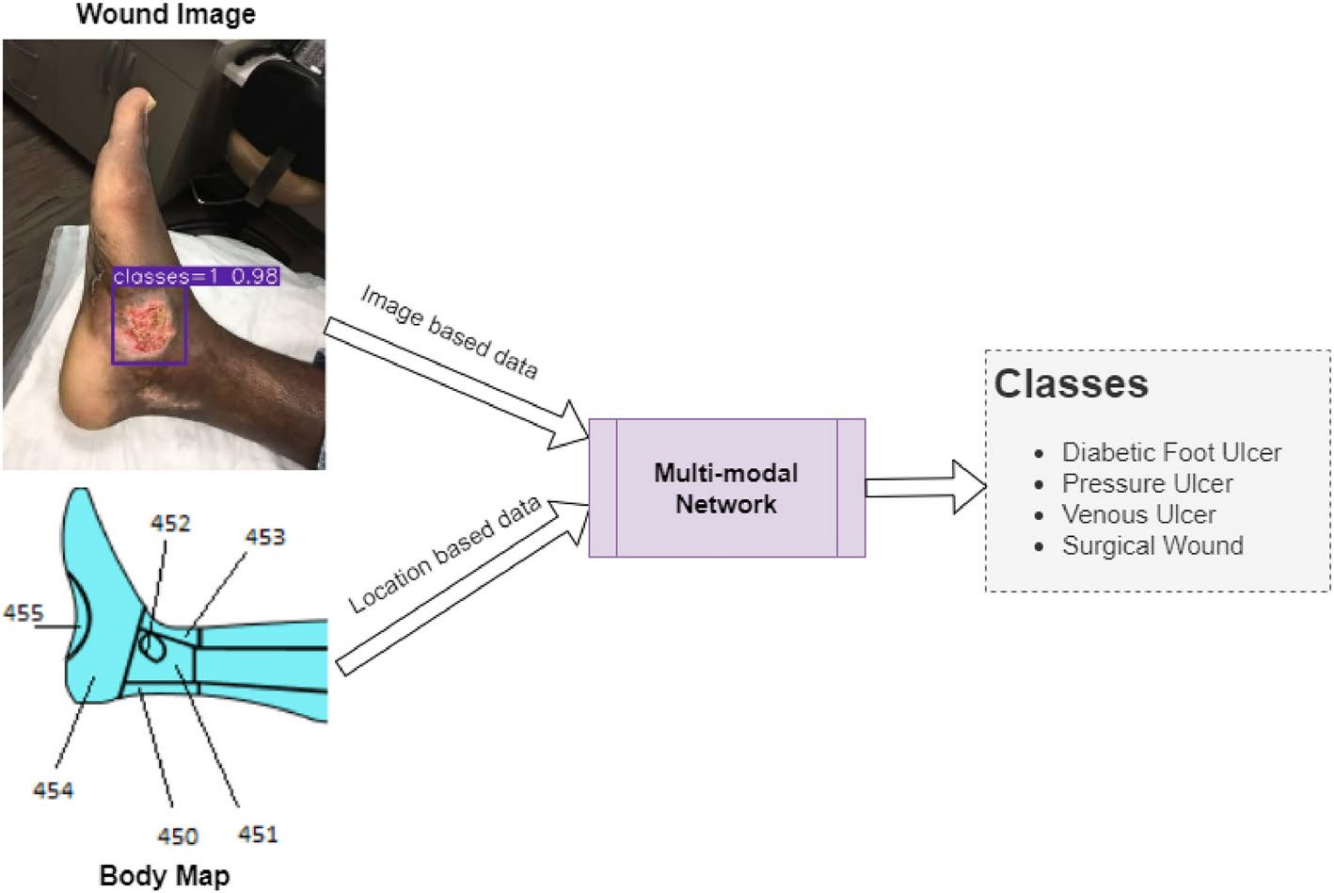
Expected workflow of this research.

A simplified workflow of this study is shown in Fig. 1. The developed wound classifier takes both wound image and location as inputs and outputs the corresponding wound class.

## Related works

In this review, we revisit the relevant research in the field of wound image classification, segmented into categories based on the methodology of each study.

### Deep learning based classification

#### Convolutional neural networks (CNNs) with SVM

A method proposed by Abubakar et al.^29^ distinguished between burn wounds and pressure ulcers using pre-trained deep architectures such as VGG-face, ResNet101, and ResNet152 in combination with an SVM for classification. Similarly, Goyal et al.^30^ predicted the presence of infection or ischemia in Diabetic Foot Ulcers (DFUs) using Faster RCNN and InceptionResNetV2 networks, in combination with SVM.

#### Advanced deep learning techniques

Advanced methods involving two-tier transfer learning were utilized in studies which used architectures like MobileNet, InceptionV2, and ResNet101. Goyal et al.^31^ presented DFUNet for classification of DFUs, while Nilsson et al.^32^ applied a CNN-based method using VGG-19 for venous ulcer image classification. In another significant study, Alaskar et al.^33^ applied deep CNNs for intestinal ulcer detection in wireless capsule endoscopy images. Using AlexNet and GoogleNet architectures, they reported a classification accuracy of 100% for both networks.

Ahsan et al.^34^ discusses the use of deep learning algorithms to automatically classify diabetic foot ulcers (DFU), a serious complication of diabetes that can lead to lower limb amputation if untreated. The authors examined various convolutional neural network (CNN) architectures, including AlexNet, VGG16, VGG19, GoogleNet, ResNet50, MobileNet, SqueezeNet, and DenseNet. They used these models to categorize infection and ischemia in the DFU2020 dataset. To address the issue of limited data and to reduce computational cost, they fine-tuned the weights of the models. Additionally, affine transform techniques were employed for data augmentation. The results revealed that the ResNet50 model achieved the highest accuracy rates, reaching 99.49% for ischemia and 84.76% for infection detection.

#### Multi-class classification techniques

Shenoy et al.^35^ proposed a method to classify wound images into multiple classes using deep CNNs. Rostami et al.^36^ proposed an ensemble DCNN-based classifier to classify entire wound images into surgical, diabetic, and venous ulcers. Anisuzzaman et al.^28^ proposed a multi-modal classifier using wound images and their corresponding locations to categorize them into multiple classes, including diabetic, pressure, surgical, and venous ulcers. This paper introduced an image and location classifier and combined it together to create a multi-modal classifier. In this study, two different datasets were used namely AZH dataset that consists of 730 wound images with four classes, Medetec dataset which consists of 358 wound images with three classes. Also, they introduced a new dataset AZHMT dataset which is a combination of AZH and Medetec dataset containing 1088 wound images. The reported maximum accuracy on mixed-class classifications varies from 82.48 to 100% in different experiments and maximum accuracy on wound-class classifications varies from 72.95 to 97.12% in various experiments.

### Wound image classification using novel approaches

Recent advancements in wound image classification have introduced innovative approaches to tackle the challenges in this domain. Alzubaidi et al.^37^ developed the DFU_QUTNet, a novel deep convolutional neural network, designed specifically to address the challenges in classifying Diabetic Foot Ulcers (DFUs). Key challenges overcome by DFU_QUTNet include the time-consuming process of collecting and professionally labeling DFU images, the difficulty in distinguishing DFUs from healthy skin due to high inter-class and intra-class variations, and the complex external boundaries and asymmetrical structure of DFUs. DFU_QUTNet addressed these issues through its unique network architecture, achieving a maximum F1-Score of 94.5% when combined with SVM classifiers. Sarp et al.^38^ introduced the XAI-CWC model, which applies explainable artificial intelligence (XAI) techniques to chronic wound classification. The model addresses the opaque nature of AI decision-making, a significant challenge in the medical field where understanding and trust in the model’s predictions are crucial. The XAI-CWC model uses transfer learning and a heatmap-based explanation mechanism to provide insights into the AI’s decision-making process. While the model shows promising results, it faces limitations due to dataset size and variation in performance across different wound types, as evidenced by varying precision, recall, and F1-scores for different categories of wounds.

### Traditional machine learning-based classification

#### SVM-based techniques

Traditional machine learning techniques have also found significant use in wound image classification. Yadav et al.^39^ used color-based feature extraction and SVM for binary classification of burn wound images. Goyal et al.^40^ used traditional machine learning and DCNN techniques for detecting and localizing DFUs, with Quadratic SVM classifiers trained on feature-rich patches extracted from the images.

Our model introduces several key improvements over existing methodologies in wound image classification, as evidenced by the comparative analysis presented in Table 1. These enhancements significantly elevate the model’s diagnostic capabilities, accuracy, and applicability in clinical settings:

1. Enhanced Classification Scope: Moving beyond the binary classification focus of previous studies^31–33,35–39^, our model is adept at performing image-wise multi-class wound type classification, offering a broader diagnostic perspective.
2. Innovative Ensemble Approach: By employing an advanced ensemble of convolutional neural networks, our model transcends the capabilities of individual networks like DFU-Net, SVM, and VGG-19^31,32,39^, demonstrating superior classification performance.
3. Integration of Anatomical Location Data: Our model uniquely incorporates anatomical location data through an Adaptive-gated MLP module, a feature not present in earlier studies, merging image and location data for comprehensive analysis.
4. Axial Attention Mechanism: The axial attention-based classifier within our model processes spatial relationships with unmatched precision, offering detailed insights beyond the capabilities of conventional methods^28^.
5. Superior Feature Extraction Techniques: Utilizing cutting-edge networks like ResNet152, VGG16, and EfficientNetb2 for feature extraction, our model accesses a richer feature set than those employed in previous research^29,30,37,40^, enhancing learning and classification accuracy.
6. Comprehensive Dataset Utilization: Our approach to using extensive and diverse datasets (AZH, Medetec) addresses and overcomes the limitations related to dataset size and diversity highlighted in previous works^28,33,35,37^.
7. Comprehensive Evaluation for Real-world Application: Our model undergoes a rigorous and comprehensive evaluation process, ensuring its effectiveness and reliability in clinical settings, a step beyond the basic metrics evaluated in prior studies^29,30,37,40^.

**Table 1:** Summary of wound image classification works.

Reference nos.	Methods	Dataset	Limitations
^31^	DFU-Net	DFU dataset: 392 images	Network struggles with small/similar-colored DFUs and normal skin with wrinkles/red tones
^39^	SVM	Burns dataset	Small evaluation or Test set (74 images)
^32^	VGG-19 network	Dataset: 300 images annotated by specialists	Accuracy varies with camera-to-ulcer distance
^33^	AlexNet and GoogleNet networks	Dataset: 1875 images	Unbalanced test set (3:1 ratio)
^35^	WoundNet (modified VGG-16) and Deepwound (ensembled network)	Dataset: 1335 wound images collected using smartphone and internet	Accuracy ranges 72%-97% across binary classes; model underperforms for specific categories such as drainage (72%)
^29^	SVM Feature extracted from VGG-face, ResNet101,152	Dataset: 29 pressure and 31 burn images	Small dataset
^37^	DFU_QUTNet	Dataset: 754 images	Evaluation limited to precision, recall, and F1-score, potentially obscuring comprehensive assessment
^30^	Features extracted using Inception-V3, ResNet50, and InceptionResNetV2 bottlenecks	Dataset: 1459 images	Model performance may decline due to lighting conditions (shadows), marks, and skin tone variations
^40^	Quadratic SVM, InceptionV2, MobileNet, ResNet101, InceptionResNetV2	Dataset: 1775 images collected from hospital	No evaluation provided for their classification task
^28^	Ensembled network using VGG16,19, ResNet50, InceptionV3 and Alexnet	Dataset: AZH 4410 images Medetec 1698 images AZHMT 6108 images (with manual augmentation)	May have poor performance due to class overlap and data scarcity, suggesting a need for larger, more diverse datasets for improved accuracy

## Materials and methods

This study encompasses three distinct subsections, each elucidating the specific methodology employed in this study: Whole Image Classification, Region of Interest (ROI) Extracted Image Classification, and ROI with Body Map Location Image Classification. It should be noted that each of these subsections utilizes the same fundamental base classifier for the image data analysis. The term fundamental base classifier is used to denote the consistent application of the same final classifier block across the different subsections of our analysis. This approach was adopted to ensure uniformity in the final classification stage, regardless of the specific preprocessing or feature extraction techniques applied in earlier stages of the model. Datasets were anonymized, partitioned, and augmented before processing through a proposed architecture. The proposed model incorporated transfer learning, convolution blocks, axial-attention mechanisms, and Adaptive-gated MLP. Model performance was evaluated using accuracy, precision, recall, and the F1-score.

### Dataset

#### AZH dataset

The AZH Dataset is a collection of prefiltered 730 ROI images and 538 Whole wound images, varying in size and depicting four types of wounds: venous, diabetic, pressure, and surgical. Captured over two years at Milwaukee’s AZH Wound and Vascular Center, the images were collected using an iPad Pro (software version 13.4.1) and a Canon SX 620 HS digital camera, and subsequently labeled by a wound specialist from the center. While most of the dataset comprises unique patient cases, some instances involve multiple images from a single patient, taken from different body sites or at varying stages of healing. These were classified as separate due to distinct wound shapes. This dataset, unfortunately, couldn’t be expanded due to resource limitations. It’s important to note that the data doesn’t involve any human experimentation or usage of human tissue samples. Instead, it utilizes de-identified wound images, available at link: https://github.com/uwm-bigdata/Multi-modal-wound-classification-using-images-and-locations. Each image only includes the wound and immediate skin area, eliminating any unnecessary or personal data to protect patient identity. The University of Wisconsin-Milwaukee has vetted the dataset’s use for compliance with university policy. Figures 4, 5 show images from whole and ROI images.

#### Medetec dataset

The Medetec wound dataset is a compendium of freely available images that encompasses an extensive range of open wounds^41^, available at link: https://www.medetec.co.uk/files/medetec-image-databases.html?. We prefiltered 216 images from three distinct categories for this study: diabetic wounds, pressure ulcers, and venous leg ulcers. Notably, this dataset does not encompass images of surgical wounds. The images are provided in .jpg format, with weights and heights fluctuating between 358 and 560 pixels, and 371 to 560 pixels, respectively. This dataset laid a solid foundation for the robustness and reliability assessments of the model we developed.

Our approach to selecting datasets was carefully planned to thoroughly evaluate our tool, with a particular focus on the AZH dataset for its broad range of wound types and detailed images. We also included the Medetec dataset, choosing only the classes that match those in the AZH dataset. This choice was informed by earlier research^28,36^ and aimed to ensure our analysis was aligned with recognized wound classifications. Such alignment allows our findings to contribute meaningfully to the field of wound image classification. We specifically chose classes from the Medetec dataset that were relevant to our research goals, acknowledging this approach’s limitations, such as excluding certain wound types. However, this was a strategic decision to enhance our tool’s effectiveness in classifying the selected wound categories. It demonstrates our commitment to precision and lays the groundwork for including a wider variety of wound types in future studies.

#### Body map for location

A body map serves as a simplified, symbolic, and accurately phenotypic representation of an individual’s body^42^. Primarily used in the medical field, body maps are effective tools for identifying and locating physical afflictions such as bruises, wounds, or fractures. They are especially valuable in forensic science for identifying bodily changes during post-mortem examinations and in medical practice for pinpointing the location of infections^43^. By offering a detailed overview of the body, they inform practitioners about other body areas that might be affected and require attention during the healing process. Furthermore, in the realm of scientific research, body maps function as verifiable evidence, validating observable bodily changes caused by internal diseases.

**Figure 2:**
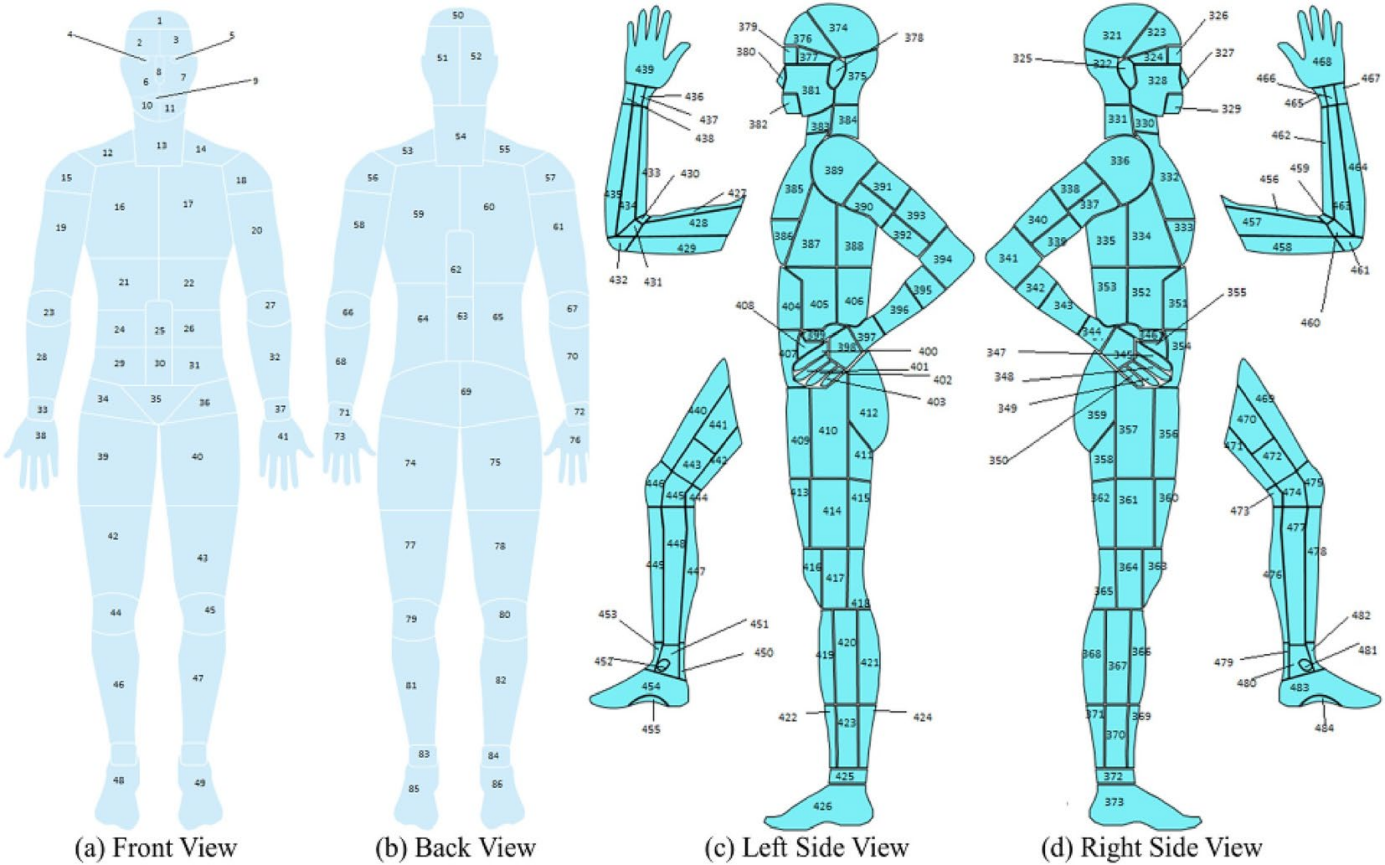
Full Body View^28^.

The design of a comprehensive body map with 484 distinct parts is credited to Anisuzzaman et al.^28^. PaintCode^44^ was employed to prepare this body map, with initial references being drawn from several credible sources^45–47^. The fundamental framework for this design originated from the Original Anatomy Mapper^48^, which directly paired each label and outline. The extreme intricacy involved in the detailed depiction of each feature on the body map led to a pre-selection of 484 features or regions. This process was overseen and approved by wound professionals at the AZH wound and vascular center, ensuring the map’s medical accuracy and applicability. Major part of body map is shown in the Fig. 2. Each number denotes a location in this case. Table 2 shows a few examples of locations and their related numbers.

**Table 2:** Body map examples of lower leg region.

Right leg front and back		Left leg front and back	
Location name	Body map number	Location name	Body map number
Right fifth toe tip	135	Left anterior ankle	180
Right lateral heel	150	Left fifth toe tip	202
Right medial malleolus	158	Left medial malleolus	178
Right proximal lateral dorsal foot	159	Left proximal medial plantar foot	215

Crucially, the alignment of location information with each wound image was meticulously verified and marked by experts at the AZH center. This ensures that the wound images used in our study are not only accurately categorized but also associated with the precise location of occurrence on the body, as delineated by the body map. This collaboration with AZH experts is instrumental in enhancing the reliability of our dataset, enabling a more accurate and clinically relevant analysis of wound locations and their implications for treatment and diagnosis.

### Dataset processing and augmentation

#### ROI extraction

The extraction of Region of Interest (ROI) from wound images presents a robust methodology for diagnosing and tracking wound progression. As aforementioned, the ROI includes the wound itself and a portion of the surroundings, which collectively encompasses the vital elements of the wound’s condition. The developed wound localizer is a significant tool for this extraction process, as it is capable of automatically cropping single or multiple ROIs from each image^49^. Each ROI represents one of six categories—diabetic, venous, pressure, surgical, background, and normal skin. These categories are critical in understanding the etiology of the wound, allowing for more accurate and personalized treatment plans. However, the diversity of the wounds is also reflected in the different sizes and shapes of the extracted ROIs, each telling a unique narrative of the wound’s journey.

Importantly, the ROI’s rectangular form and variable size allow for greater adaptability in handling various wound types and sizes. It is an efficient method to focus on the essential wound characteristics while reducing unnecessary information that could potentially introduce noise into the data. Figure 5 excellently illustrates the variation in extracted ROIs from different classes of the wound dataset. This showcases the versatility of our wound localizer, capable of handling wounds of different origins, sizes, and stages. It successfully extracts the ROI, making the most relevant information available for analysis.

**Figure 3:**
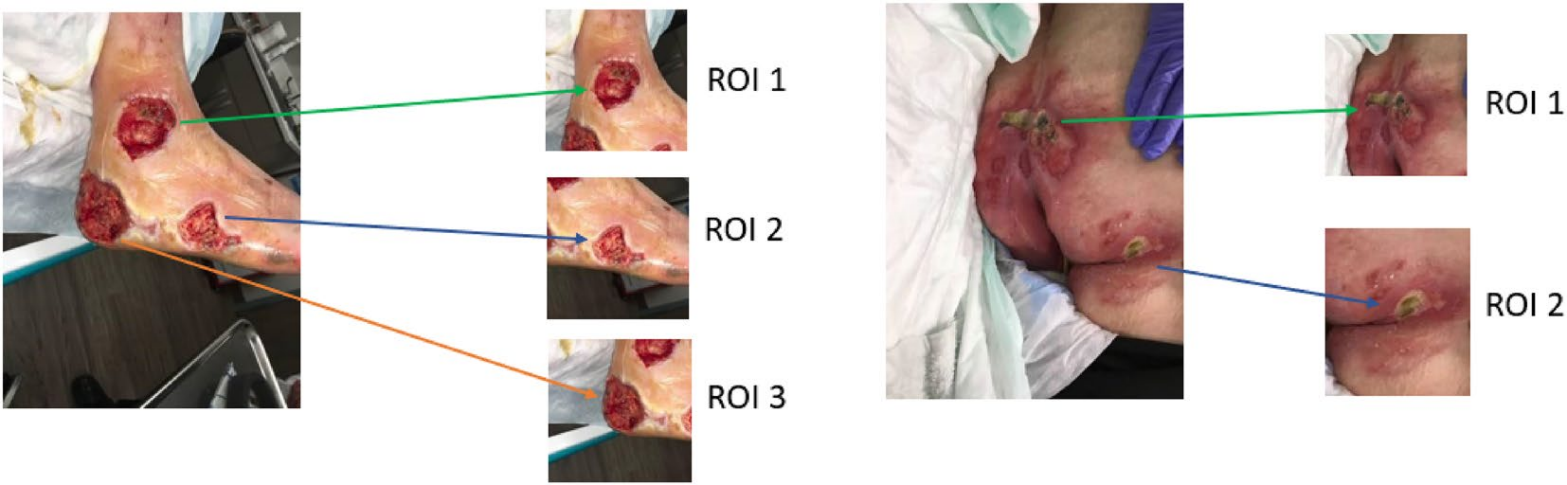
ROI Extraction and overlapping.

In the context of multi-class image classification, Fig. 3 highlights a phenomenon where wound regions may overlap within the extracted ROIs. It is pertinent to note, however, that such overlaps are between regions belonging to the same class category. Therefore, this overlapping should not adversely impact the model’s ability to classify wounds correctly, as the central, most clinically significant portion of the wound is consistently positioned at the center of the image when the ROI is cropped. This centric approach to ROI cropping ensures that the most critical region for classification remains the focal point, further reinforcing the model’s classification accuracy despite any peripheral overlap in the images.

#### Data split

**Figure 4:**
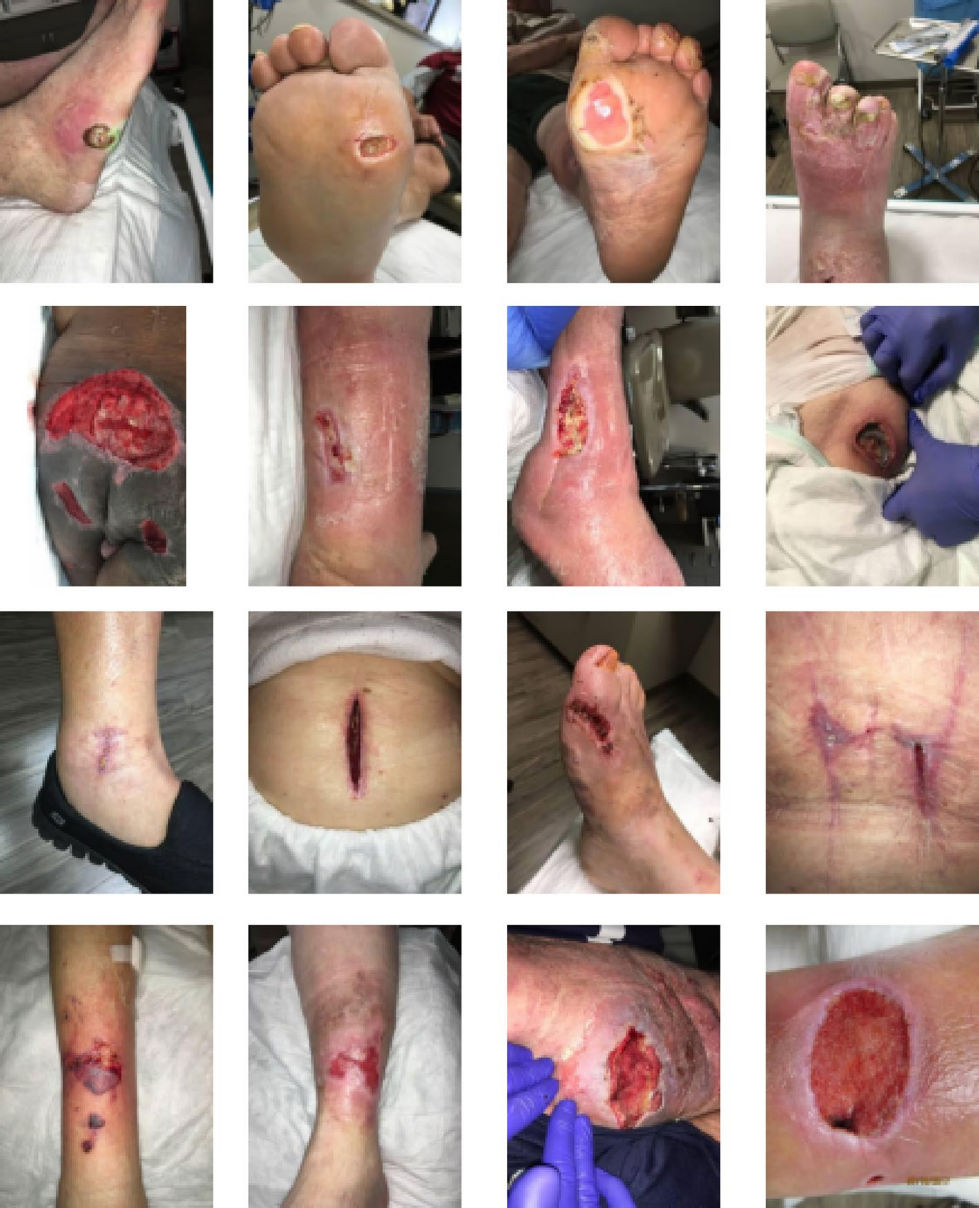
Sample images from the AZH Wound and Vascular Center database. The rows from top to bottom display diabetic, pressure, surgical and venous samples, respectively.

**Figure 5:**
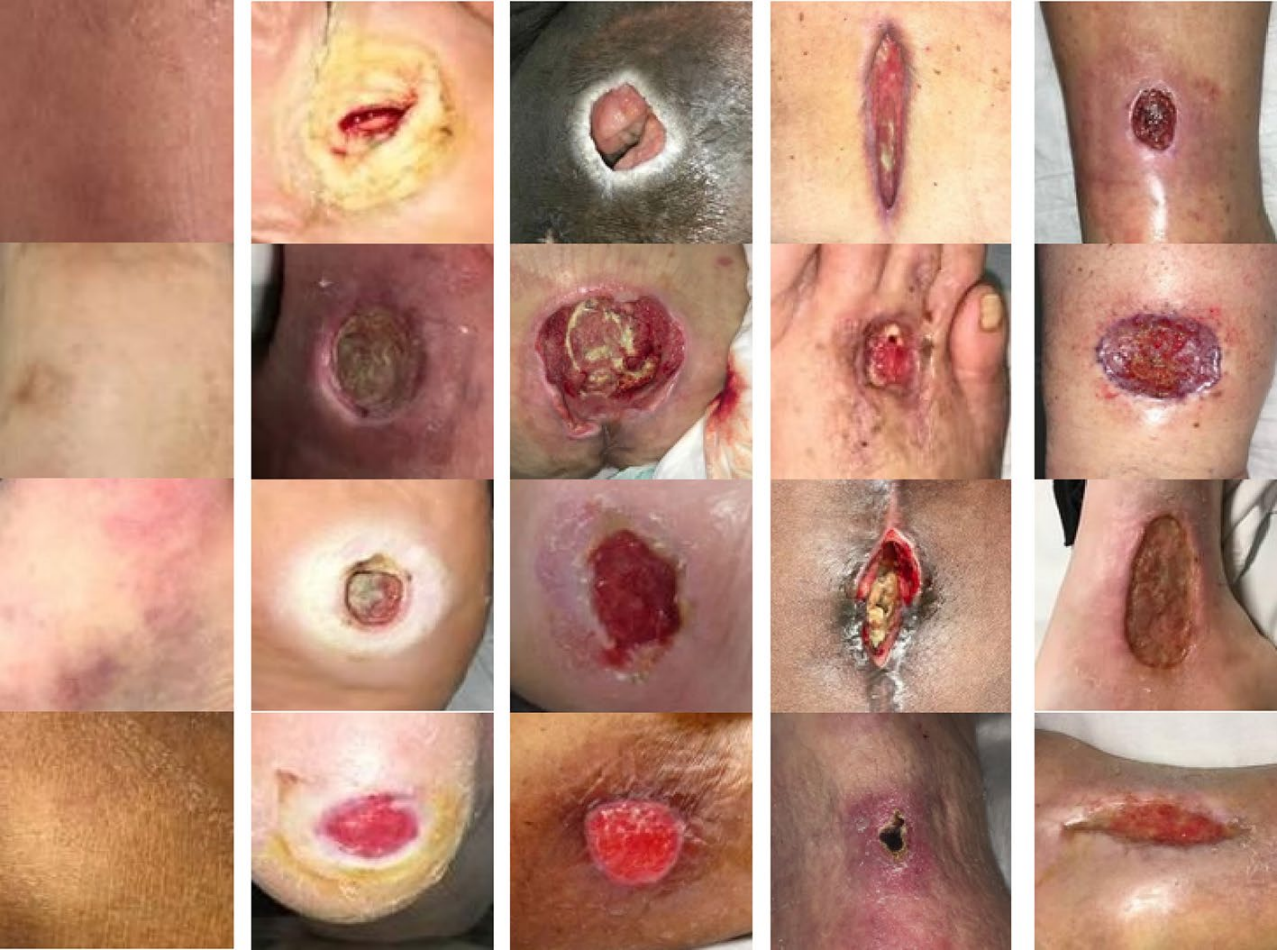
The columns from left to right display normal skin, diabetic, pressure, surgical and venous ROIs, respectively.

During this study, we implemented two distinct methods for partitioning the dataset, aiming to gain a deeper understanding of the model’s behavior, uncover potential biases and sensitivities, and assess its generalization capabilities. This dual approach to dataset partitioning enables a more robust and comprehensive evaluation process. Initially, the data was split into training (70%), testing (15%), and validation (15%) subsets. A second partitioning strategy slightly modified these proportions, allocating 60% of the data to training, maintaining 15% for validation, and expanding the testing set to encompass 25% of the total dataset, as detailed in Table 3. This strategic distribution between training, validation, and testing sets is pivotal in calibrating the model’s performance. The training set is essential for model learning, introducing a wide array of examples from which the model can generalize. The validation set, crucial for tuning the model’s hyperparameters, provides a feedback loop for performance optimization without compromising the integrity of the test set. This iterative refinement process ensures that adjustments are made to enhance the model’s accuracy and generalizability. Finally, the testing set offers a critical assessment of the model’s predictive capability on unseen data, serving as the definitive benchmark for evaluating real-world applicability. By ensuring that images from the same wound are exclusively allocated to one subset, we safeguard against data leakage and maintain the validity of our evaluation process (Figs. 4, 5).

**Table 3:** AZH and Medetec original dataset count and class abbreviations.

Class abbreviation		Back ground	Normal Skin	Venous	Diabetic	Pressure	Surgical	Sub Total	Total
		BG	N	V	D	P	S		
ROI image-based dataset: 60% training, 15% validation, 25% testing									
AZH	Training	60	60	148	111	80	98	557	930
	Validation	15	15	37	27	20	24	138	
	Testing	25	25	62	47	34	42	235	
ROI image-based dataset: 70% training, 15% validation, 15% testing									
AZH	Training	70	70	172	129	93	114	648	930
	Validation	15	15	37	27	20	24	138	
	Testing	15	15	38	29	21	26	144	
Whole image-based dataset: 60% training, 15% validation, 25% testing									
AZH	Training	–	–	93	92	60	76	321	538
	Validation	–	–	23	23	15	19	80	
	Testing	–	–	40	39	25	33	137	
Whole image-based dataset: 70% training, 15% validation, 15% testing									
AZH	Training	–	–	109	107	70	89	375	538
	Validation	–	–	23	23	15	19	80	
	Testing	–	–	24	24	15	20	83	
Whole image-based dataset: 60% training, 15% validation, 25% testing									
Medetec	Training	–	–	37	27	65	–	129	216
	Validation	–	–	9	6	16	–	31	
	Testing	–	–	16	12	28	–	56	
Whole image-based dataset: 70% training, 15% validation, 15% testing									
Medetec	Training	–	–	43	31	76	–	150	216
	Validation	–	–	9	6	16	–	31	
	Testing	–	–	10	8	17	–	35

#### Data augmentation

**Figure 6:**
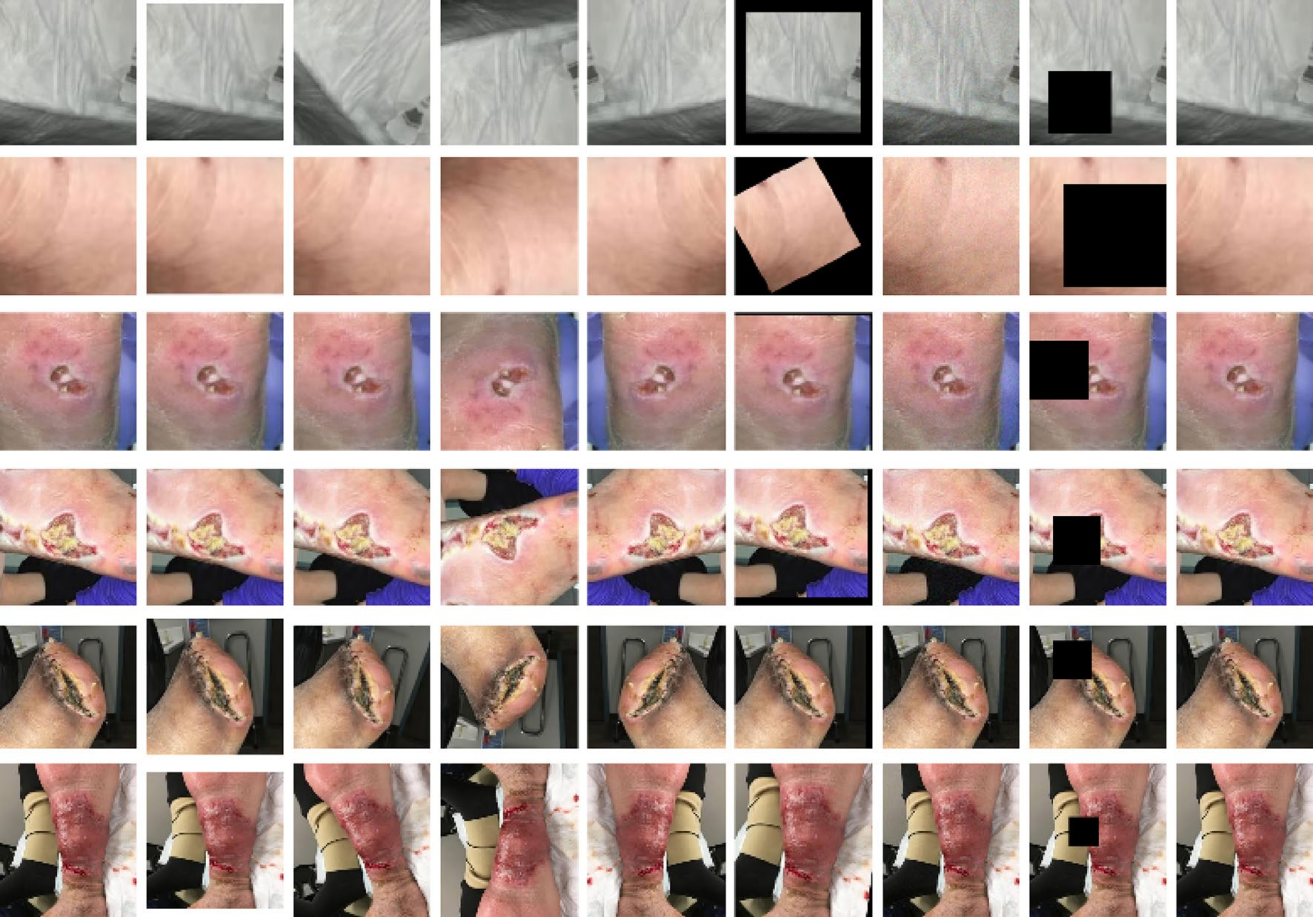
Data Augmentation with leftmost original image. The rows from top to bottom display background, normal skin, diabetic, pressure, surgical and venous ROIs, respectively.

Each image in the training set was augmented using transformation methods such as resizing, rotation, flipping (both vertically and horizontally), and application of affine transforms (including scaling, rotating, and shifting). Additional alterations such as the application of Gaussian noise and coarse dropout (i.e., random rectangular region removal) were also performed. These transformations were probabilistically applied, creating a diverse set of augmented samples as shown in Fig. 6. The transformations ensured robustness of the model against variations in the data.

#### ROI and wound location

In the ROI dataset we have two additional classes named normal skin and background which were created manually by selecting skin region for normal skin and any additional information as background from the original whole image dataset^28^. Sample of these two classes are shown in Fig. 5. All of these were verified by wound specialists. Wound location was associated with each ROI image and assigned values from the body map discussed in section B. All the six classes abbreviation is shown in Table 3.

### Model

**Figure 7:**
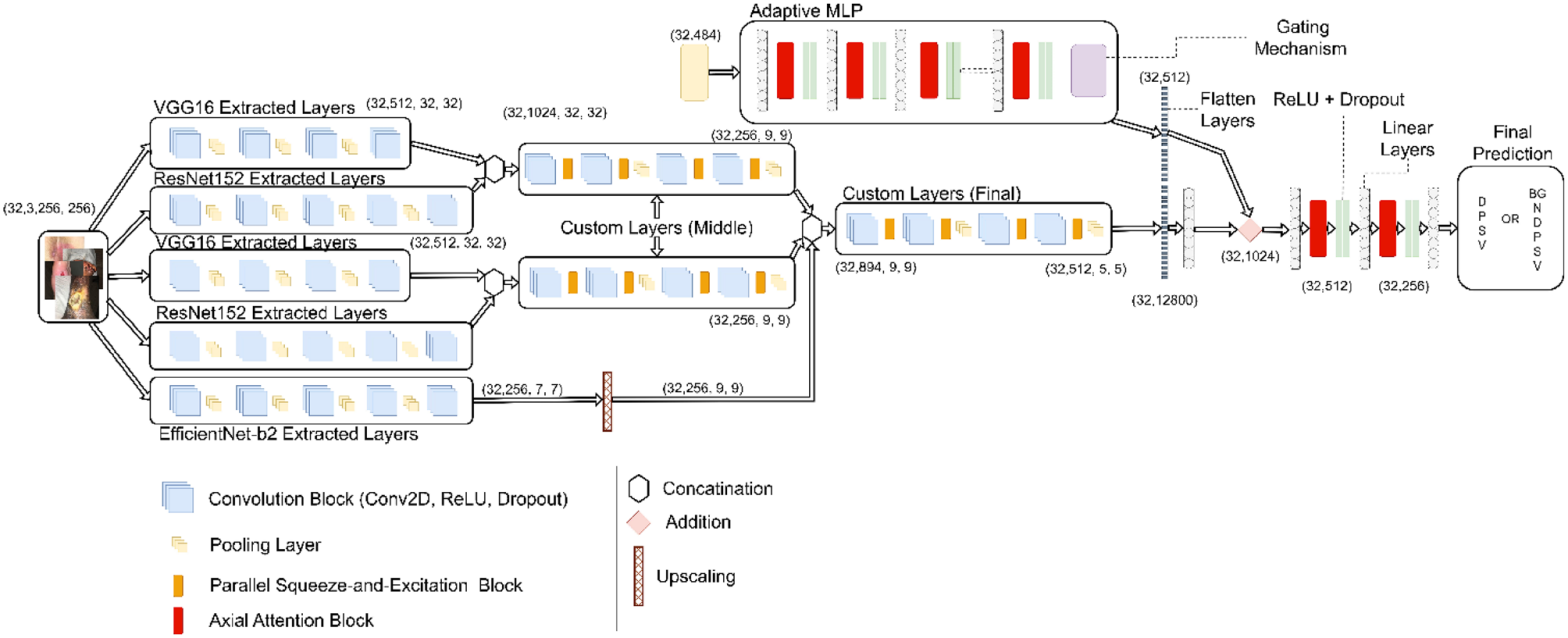
Proposed model architecture outline.

Our proposed deep learning model integrates multi-level features from various pre-existing models, utilizing custom layers and attention mechanisms to improve performance. Our model design has been adopted from C-Net architecture^50,51^. Basic model outline is displayed in Fig. 7.

#### Base models

The proposed model utilizes three pre-trained Convolutional Neural Networks (CNNs)—ResNet152, VGG16, and EfficientNet-B2. In ResNet152, modifications include not only the removal of the average pooling and fully connected layers, but also alterations to the third block of the second layer by removing its last two layers and removing the final four layers of the overall model. For VGG16, the last twelve layers are omitted, capturing more primitive patterns. The last layer of EfficientNet-B2 is removed to maintain consistency with the modifications made to the other two models. These models, applied in parallel to the input, capture different levels of features.

#### Custom layers

The custom layers comprise a Convolutional Block (ConvBlock), followed by a Parallel Squeeze-and-Excitation (P_scSE) block^52^, and a dropout layer. The ConvBlock is a combination of a convolution layer and a ReLU activation function, capturing spatial information and introducing non-linearity.

**Figure 8:**
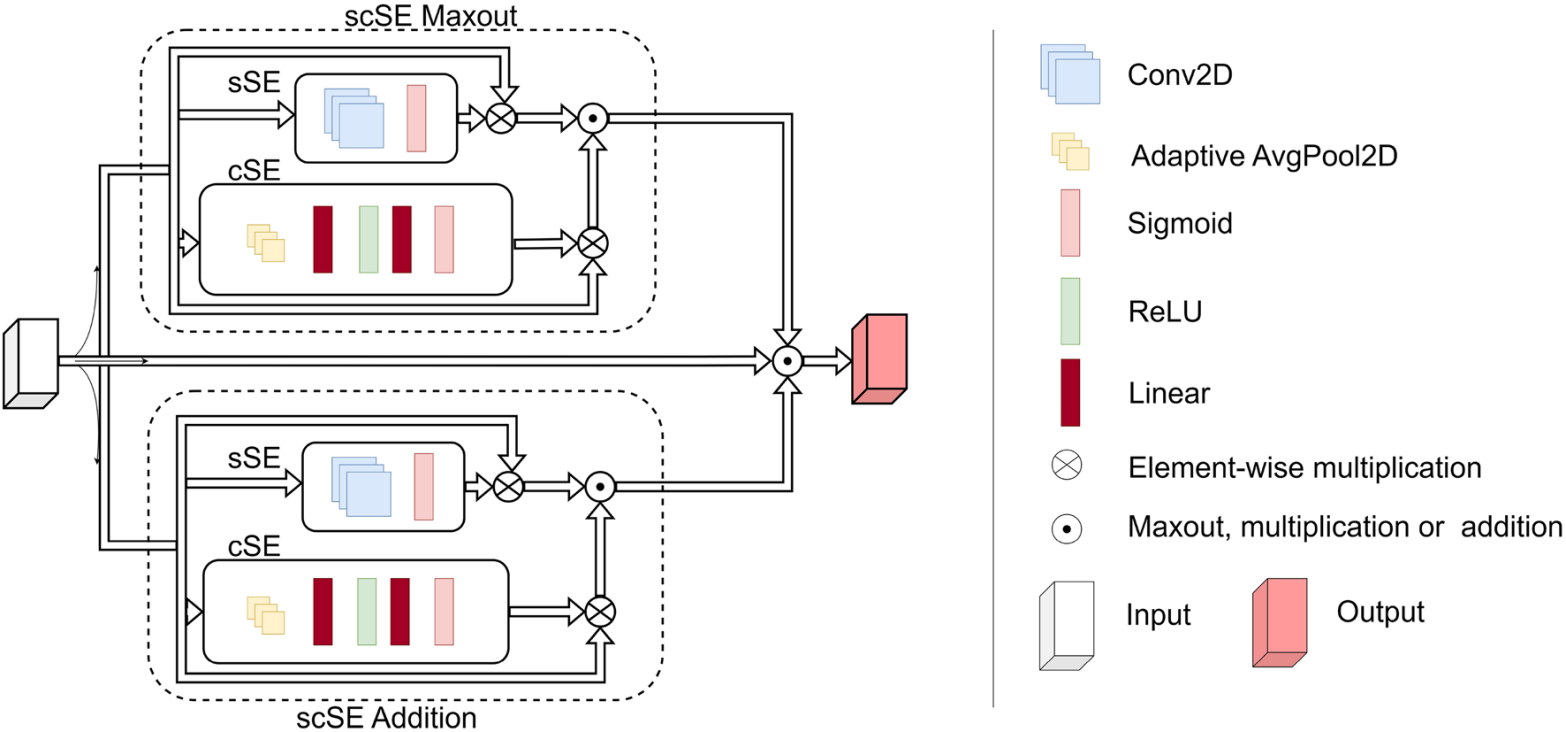
Parallel Squeeze-and-Excitation block architecture outline.

The P_scSE block blends Channel-wise Squeeze-and-Excitation (cSE) and Spatial Squeeze-and-Excitation (sSE) operations. The cSE focuses on channel interdependencies, providing global context, while the sSE concentrates on the spatial interdependencies of each channel, maintaining detailed spatial information. Outputs from the cSE and sSE are merged using max-out and addition operations^52^ as shown in Fig. 8. The integration of P_scSE blocks serves a dual purpose. The cSE is instrumental in capturing global contextual information by focusing on channel interdependencies. This aspect is crucial for recognizing patterns significant across different channels. Conversely, the sSE is tailored to maintain high-resolution spatial information by concentrating on spatial interdependencies within each channel. The combination of these two operations allows our model to extract and utilize both global and local features more effectively, thereby enhancing its classification capabilities for wound images with intricate details.

#### Aggregation and fully connected layers

The base models’ outputs are concatenated and fed through sequences of ConvBlocks and P_scSE blocks to merge and process multi-level features. The output is then flattened and passed through a dense layer.

The output is further processed through a fully connected layer block. This block includes two dense layers enriched with axial-attention mechanisms, an enhancement over traditional attention mechanisms, focusing on individual dimensions separately. Interspersed with ReLU activation functions and dropout operations, the axial-attention mechanisms in our model are designed to process one dimension of data at a time, unlike traditional attention mechanisms that operate on the entire feature map. This dimension-wise processing makes the axial-attention more efficient and potent in capturing long-range dependencies within the image data. By focusing on individual dimensions separately, the axial-attention mechanism elevates the model’s ability to discern complex patterns and dependencies, a feature particularly advantageous for the detailed task of wound classification.

The inclusion of the Adaptive-gated Multi-Layer Perceptron (MLP) in our model is specifically to handle the wound location data, which differs significantly in nature from image data. This module processes the wound location data separately, using a series of linear transformations and axial attentions. The gating mechanism in the MLP is a pivotal feature; it enables the model to learn and selectively propagate only relevant location information. This selective propagation ensures that the integration of location data into the image analysis enhances the model’s classification accuracy, making it robust against irrelevant or misleading information.

The orderly arrangement of data is vital for the efficient functioning of the model. Consistency in the output from the image and location data is essential, thus necessitating the synchronous feeding of properly sequenced data into the model. This alignment was maintained by associating each Region of Interest (ROI) with a unique index number and mapping the corresponding wound location to this number. Given the categorical nature of wound location data, it was represented using one-hot encoding.

#### Output layer

The final dense layer maps to the number of output classes.

### Performance metrics

In our study, we employed various evaluation metrics such as accuracy, precision, recall, and the F1-score to scrutinize the effectiveness of the classifiers. For a more comprehensive understanding of these equations and associated theories, readers are referred to reference^28,36,53^.

## Results

In the present investigation, we deployed the advanced computational capacities of Google Colab Pro Plus A100, fortified with 40 GB of memory. This enabled a methodical analysis involving both Region of Interest (ROI) and whole image-based classifications. The experimental setup involved processing images of 256 × 256 pixel dimensions, batched into groups of 32, across a course of 100 epochs. Our learning parameters were finely tuned to optimize the learning process: a learning rate of 0.0001 was chosen, with a minimum rate limit of 0.00001. To enhance the efficiency of our learning process, we applied the Adam optimizer^54^.

*Classification categories* The classifiers were extensively trained to distinguish among various classes represented in the images, specifically: Diabetic (D), Venous (V), Pressure (P), Surgical (S), Background (BG), and Normal Skin (N). Further specifications and results regarding these classes will be provided in the ensuing sections of this paper.

*Loss function* Cross Entropy was chosen as our loss function, given the multi-class and binary nature of our image classifications. Its mathematical formulation is as follows^35,55^:

For multi-class problems, the cross-entropy loss, L, is (Eq. 1):

1 $${\text{L}} = - \mathop \sum \limits_{{{\text{i}} = 0}}^{{\text{n}}} \left( {{\text{y}}_{{\text{i}}} \times {\text{log}}\left( {{\text{p}}_{{\text{i}}} } \right)} \right)$$

Here y_i_ is the actual label and (p_i_) is the predicted probability for each class (i). For binary classification problem, the binary cross entropy loss, L, is computed as (Eq. 2):

2 $${\text{L}} = - \mathop \sum \limits_{{{\text{i}} = 0}}^{{\text{n}}} \left( {{\text{y}}_{{\text{i}}} \times \log \left( {{\text{p}}_{{\text{i}}} } \right) + \left( {1 - {\text{y}}_{{\text{i}}} } \right) \times \log \left( {1 - {\text{p}}_{{\text{i}}} } \right)} \right)$$

The optimization process strives to minimize this loss, thereby reducing the discrepancy between our model’s predictions (p_i_) and the actual labels (y_i_). Further sections will elucidate the efficacy of this loss function within our research context.

### ROI classification

The primary phase of the ROI classification trial pertains to the classification of 6 unique types of wound patches, specifically: diabetic, venous, pressure, surgical, BG, and N. Subsequently, the 5-category classification problem comprised three types of wound labels alongside BG and N categories. When addressing the 4-category classification, the objective centered on the categorization of the wound patches into one of the four classes: BG, N, along with two different wound labels. In the context of 3-category classification, the aim was to sort the wound patches into one of the three groups: D, P, S, V. For binary classification, a range of combinations including N, D, P, S, V were utilized to categorize the wound patches into two distinct groups. The dataset was split in two different ways, one is 70-15-15 and the other is 60-15-25, to observe and compare the best results.

#### ROI multiclass classification without wound location

The results of the ROI classifier’s performance without wound location evaluation varied across different scenarios. For the 6-class classification case (BG, N, D, P, S, V), the test accuracy was 85.41% and 80.42% for the 70%, 15%, 15% and 60%, 15%, 25% data splits respectively. The precision, recall and F1-score for this case were 85.69%, 85.41%, 85.29% and 80.26%, 80.42%, 79.52% for each data split respectively, as displayed in Table 4.

**Table 4:** ROI image without location-based classification with different data split (Left—70%, 15%, 15%, Right—60%, 15%, 25%).

No. of classes	Classes	A	P	R	F	No. of classes	Classes	A	P	R	F
6 class	BG, N, D, P, S, V	**85.41**	85.69	85.41	85.29	6 class	BG, N, D, P, S, V	**80.42**	80.26	80.42	79.52
5 class	BG, N, D, P, V	88.13	89.01	88.13	87.80	5 class	BG, N, D, P, V	83.41	83.34	83.41	83.20
	BG, N, D, S, V	**91.86**	92.29	91.86	91.91		BG, N, D, S, V	**91.04**	91.30	91.04	90.96
	BG, N, D, P, S	87.73	88.91	87.73	87.74		BG, N, D, P, S	84.39	84.39	84.39	84.39
	BG, N, P, S, V	86.95	86.83	86.95	86.56		BG, N, P, S, V	87.23	87.25	87.23	87.15
4 class	BG, N, D, V	**96.90**	97.04	96.90	96.90	4 class	BG, N, D, V	**96.22**	96.31	96.22	96.23
	BG, N, P, V	95.50	95.57	95.50	95.48		BG, N, P, V	91.09	91.21	91.09	90.96
	BG, N, S, V	94.68	94.76	94.68	94.69		BG, N, S, V	91.55	91.68	91.55	91.51
	BG, N, D, P	87.50	87.58	87.50	87.47		BG, N, D, P	87.02	87.43	87.02	87.16
	BG, N, D, S	90.58	91.20	90.58	90.59		BG, N, D, S	89.2	91.81	89.20	89.06
	BG, N, P, S	87.01	89.16	87.01	87.30		BG, N, P, S	85.71	85.88	85.71	85.78
3 class	D, S, V	**91.39**	91.93	91.39	91.30	3 class	D, S, V	**90.72**	90.98	90.72	90.49
	P, S, V	87.05	87.18	87.05	87.10		P, S, V	83.33	83.26	83.33	83.01
	D, P, S	82.89	83.64	82.89	82.41		D, P, S	74.79	74.28	74.79	74.39
	D, P, V	86.36	85.99	86.36	85.75		D, P, V	85.31	85.20	85.31	84.71
2 class	N, D	**100.0**	100.0	100.0	100.0	2 class	N, D	**100.0**	100.0	100.0	100.0
	N, P	94.44	95.09	94.44	94.47		N, P	96.61	96.61	96.61	96.61
	N, S	**100.0**	100.0	100.0	100.0		N, S	98.5	98.54	98.50	98.50
	N, V	98.11	98.23	98.11	98.13		N, V	**100.0**	100.0	100.0	100.0
	D, P	88.00	88.00	88.00	88.00		D, P	85.18	87.05	85.18	84.62
	D, S	90.90	91.35	90.90	90.85		D, S	89.88	90.34	89.88	89.82
	D, V	98.50	98.55	98.50	98.51		D, V	97.24	97.27	97.24	97.25
	P, S	85.10	85.23	85.10	85.13		P, S	81.57	81.56	81.57	81.51
	P, V	93.22	93.29	93.22	93.13		P, V	90.62	90.61	90.62	90.51
	S, V	93.75	93.85	93.75	93.70		S, V	93.26	93.25	93.26	93.25

*P* precision, *R* recall, *F* F1-score, *A* accuracy. Significant values are in bold.

In the 5-class classification scenario, the results varied between the class combinations. The BG, N, D, S, V combination showed superior performance with test accuracies, precisions, recalls, and F1-scores of 91.86%, 92.29%, 91.86%, 91.91% and 91.04%, 91.30%, 91.04%, 90.96% for each data split respectively. Conversely, the BG, N, D, P, S class combination registered slightly lower accuracy rates of 87.73% and 84.39%, along with precision, recall and F1-score values of 88.91%, 87.73%, 87.74% and 84.39%, 84.39%, 84.39% for each data split respectively.

When the classifier was tested for 4-class classification, BG, N, D, V demonstrated high accuracy rates of 96.90% and 96.22%, with precision, recall, and F1-score of 97.04%, 96.90%, 96.90% and 96.31%, 96.22%, 96.23% for each data split respectively. However, the BG, N, P, S combination indicated a decrease in accuracy at 87.01% and 85.71%, along with precision, recall, and F1-score values of 89.16%, 87.01%, 87.30% and 85.88%, 85.71%, 85.78% for each data split respectively.

The performance for 3-class and 2-class classification showed a range of accuracy scores, with the 2-class case achieving 100% accuracy for the N, D combination in both data splits, with corresponding precision, recall, and F1-score values also being 100%.

Data augmentation is applied exclusively to the training set to enhance the model’s generalization by introducing a broader range of variations, thereby preventing overfitting. This approach ensures the model learns from a diverse dataset, improving its predictive performance on unseen data. The validation and testing sets remain un-augmented to accurately assess the model’s ability to generalize to new, unmodified examples, providing a true measure of its performance in real-world scenarios. This distinction is crucial for evaluating the effectiveness and robustness of the model in practical applications.

#### ROI multi-class classification with wound location

Following the inclusion of wound location data in conjunction with image data, Table 5 displays the performance metrics from experiments using an Adaptive-gated MLP to separately analyze the wound location. This data was subsequently concatenated with the fully connected layers of the prior model.

**Table 5:** ROI image with location-based classification with different data split (Left—70%, 15%, 15%, Right—60%, 15%, 25%).

No. of classes	Classes	A	P	R	F	No. of classes	Classes	A	P	R	F
6 class	BG, N, D, P, S, V	**87.50**	88.04	87.50	87.37	6 class	BG, N, D, P, S, V	**83.82**	83.42	83.82	83.53
5 class	BG, N, D, P, V	91.52	91.51	91.52	91.44	5 class	BG, N, D, P, V	89.11	88.88	89.11	88.71
	BG, N, D, S, V	**91.86**	91.99	91.86	91.85		BG, N, D, S, V	**91.54**	91.65	91.54	91.50
	BG, N, D, P, S	84.90	85.28	84.90	84.96		BG, N, D, P, S	84.39	85.56	84.39	83.92
	BG, N, P, S, V	86.70	86.99	86.70	86.34		BG, N, P, S, V	88.82	88.81	88.82	88.70
4 class	BG, N, D, V	95.87	96.06	95.87	95.83	4 class	BG, N, D, V	**96.22**	96.37	96.22	96.24
	BG, N, P, V	94.38	94.50	94.38	94.34		BG, N, P, V	93.15	93.52	93.15	93.10
	BG, N, S, V	**96.80**	97.04	96.80	96.80		BG, N, S, V	96.10	96.19	96.10	96.05
	BG, N, D, P	88.75	89.05	88.75	88.78		BG, N, D, P	89.31	90.50	89.31	89.06
	BG, N, D, S	92.94	93.24	92.94	92.74		BG, N, D, S	93.52	93.72	93.52	93.57
	BG, N, P, S	90.90	91.50	90.90	91.03		BG, N, P, S	88.88	88.90	88.88	88.72
3 class	D, S, V	**92.47**	92.79	92.47	92.34	3 class	D, S, V	**90.72**	91.23	90.72	90.64
	P, S, V	87.05	87.18	87.05	87.10		P, S, V	84.05	83.87	84.05	83.38
	D, P, S	81.57	81.69	81.57	81.07		D, P, S	73.98	76.52	73.98	71.58
	D, P, V	89.77	90.04	89.77	89.58		D, P, V	86.71	86.53	86.71	86.47
2 class	N, D	**100.0**	100.0	100.0	100.0	2 class	N, D	**100.0**	100.0	100.0	100.00
	N, P	94.44	95.09	94.44	94.47		N, P	96.61	96.61	96.61	96.61
	N, S	97.56	97.65	97.56	97.54		N, S	98.50	98.54	98.50	98.50
	N, V	98.11	98.16	98.11	98.09		N, V	98.85	98.89	98.85	98.85
	D, P	86.14	86.14	86.00	86.03		D, P	86.41	86.73	86.41	86.22
	D, S	92.72	92.97	92.72	92.70		D, S	89.88	90.34	89.88	89.82
	D, V	98.50	98.55	98.50	98.51		D, V	97.24	97.27	97.24	97.25
	P, S	87.23	87.61	87.23	87.26		P, S	84.21	84.39	84.21	84.24
	P, V	96.61	96.77	96.61	96.56		P, V	92.70	92.99	92.70	92.56
	S, V	95.31	95.65	95.31	95.25		S, V	94.23	94.42	94.23	94.17

*P* precision, *R* recall, *F* F1-score, *A* accuracy. Significant values are in bold.

For the 6-class classification comprising BG, N, D, P, S, and V classes, the accuracy was recorded at 87.50% and 83.82%, precision at 88.04% and 83.42%, recall at 87.50% and 83.82%, and F1-score at 87.37% and 83.53% for the data splits of 70%,15%,15% and 60%,15%,25% respectively.

Moving on to the 5-class classification, the class combination BG, N, D, S, V saw strong results with accuracy levels of 91.86% and 91.54%, precision at 91.99% and 91.65%, recall at 91.86% and 91.54%, and F1-score at 91.85% and 91.50% across the two data splits. Conversely, the BG, N, D, P, S combination demonstrated lower accuracy at 84.90% and 84.39%, precision at 85.28% and 85.56%, recall at 84.90% and 84.39%, and F1-score at 84.96% and 83.92%.

In the context of the 4-class classification, the BG, N, D, V combination once again showed impressive metrics with accuracy rates of 95.87% and 96.22%, precision at 96.06% and 96.37%, recall at 95.87% and 96.22%, and F1-score at 95.83% and 96.24%. On the other hand, the BG, N, P, S combination witnessed a decrease in performance, registering accuracy levels of 90.90% and 88.88%, precision at 91.50% and 88.90%, recall at 90.90% and 88.88%, and F1-score at 91.03% and 88.72% for each respective data split.

For the 3-class and 2-class classification models, a range of performance scores were observed. The 2-class case, particularly the N, D combination, achieved perfect performance with accuracy, precision, recall, and F1-score all at 100% in both data splits. The D, P class combination, however, recorded the lowest performance levels for this category with accuracy at 86.14% and 86.41%, precision at 86.14% and 86.73%, recall at 86.00% and 86.41%, and F1-score at 86.03% and 86.22%.

In conclusion, the results show that the incorporation of wound location data alongside image data led to variations in accuracy, precision, recall, and F1-score based on the number and combination of classes, as well as the distribution of the data split. Furthermore, the use of an Adaptive-gated MLP for separate wound location analysis consistently resulted in promising outcomes across all experiments.

### Whole image classification

In the whole image classification, the precision, recall, and F1-score measurements show that the incorporation of these metrics, alongside accuracy, provides a more comprehensive understanding of the model’s performance. Table 6 depicts these additional measurements, and they reveal interesting patterns that match with the observed accuracy rates.

**Table 6:** AZH Whole image-based classification with different data split (Left—70%, 15%, 15%, Right—60%, 15%, 25%).

No. of classes	Classes	A	P	R	F	No. of classes	Classes	A	P	R	F
4 class	D, P, S, V	**83.13**	83.22	83.13	82.26	4 class	D, P, S, V	**78.10**	78.60	78.10	76.75
3 class	D, S, V	**92.64**	93.48	92.64	92.54	3 class	D, S, V	**87.50**	88.22	87.50	87.48
	P, S, V	89.83	89.71	89.83	89.73		P, S, V	85.71	85.80	85.71	85.71
	D, P, S	81.35	82.66	81.35	80.72		D, P, S	76.28	76.65	76.28	74.87
	D, P, V	87.30	87.30	87.30	87.30		D, P, V	82.69	82.52	82.69	82.30
2 class	N, D	**100.0**	100.00	100.00	100.00	2 class	N, D	**100.0**	100.00	100.00	100.00
	N, P	**100.0**	100.00	100.00	100.00		N, P	**100.0**	100.00	100.00	100.00
	N, S	**100.0**	100.00	100.00	100.00		N, S	**100.0**	100.00	100.00	100.00
	N, V	**100.0**	100.00	100.00	100.00		N, V	**100.0**	100.00	100.00	100.00
	D, P	87.17	89.38	87.17	86.50		D, P	84.37	86.03	84.37	83.61
	D, S	95.45	95.80	95.45	95.42		D, S	95.83	96.13	95.83	95.81
	D, V	**100.0**	100.00	100.00	100.00		D, V	96.20	96.23	96.20	96.20
	P, S	88.57	89.26	88.57	88.62		P, S	82.75	83.07	82.75	82.82
	P, V	89.74	91.20	89.74	89.34		P, V	89.23	89.34	89.23	89.08
	S, V	95.45	95.80	95.45	95.42		S, V	95.89	96.23	95.89	95.89

*P* precision, *R* recall, *F* F1-score, *A* accuracy. Significant values are in bold.

For the 4-class classification comprising D, P, S, and V, precision, recall, and F1-scores were observed at 83.22%, 83.13%, and 82.26% respectively for the 70–15-15 data split. For the 60–15-25 split, these scores were slightly lower, coming in at 78.60%, 78.10%, and 76.75%, respectively. This pattern is similarly reflected in the accuracy measurements for the same class combination and data splits (Table 7).

**Table 7:** Medetec Whole image-based classification with different data split (Left—70%, 15%, 15%, Right—60%, 15%, 25%).

No. of classes	Classes	A	P	R	F	No. of classes	Classes	A	P	R	F
3 class	D, P, V	**88.57**	88.65	88.57	88.50	3 class	D, P, V	**87.50**	87.44	87.50	87.43

*P* precision, *R* recall, *F* F1-score, *A* accuracy. Significant values are in bold.

In the 3-class classification, the D, S, V combination showed a high precision of 93.48%, recall of 92.64%, and F1-score of 92.54% for the 70-15-15 split. Conversely, the D, P, S combination demonstrated lower values, with a precision of 82.66%, recall of 81.35%, and F1-score of 80.72% in the same split.

Focusing on the 2-class classification, all N-related combinations (N, D; N, P; N, S; N, V) achieved perfect precision, recall, and F1-score of 100% in both data splits. However, other combinations like D, P and P, S displayed lower scores. The D, P combination, for instance, recorded precision, recall, and F1-score of 89.38%, 87.17%, and 86.50% respectively for the 70-15-15 split, and 86.03%, 84.37%, and 83.61% respectively for the 60-15-25 split.

In conclusion, the whole image classification performance, as depicted by precision, recall, F1-score, and accuracy, varies based on the number of classes and the specific class combinations. N-related combinations in the 2-class category consistently showed perfect precision, recall, and F1-scores, indicating optimal classification performance. These results provide significant insights and avenues for further research and optimization in whole image classification.

### Cross validation

Cross-validation is a robust methodology we employed in our experiments to validate the performance of our machine learning model. It involves splitting the data into several subsets or folds, in this case, five. We then train the model on all but one of these folds, testing the model’s performance on the unused fold as displayed in Table 8. This process is repeated for each fold, giving us a better understanding of how our model might perform on unseen data. It’s particularly useful in situations where our dataset is limited, as it maximizes the use of data for both training and validation purposes. Due to resource constraints, our experimental scope was confined to select procedures. As such, we were only able to conduct a limited subset of experiments, focusing on those deemed most crucial and promising.

**Table 8:** Cross Validation performed on randomly selected classes for each of the above experiment.

No. of classes	Classes	Fold1	Fold2	Fold3	Fold4	Fold5	AVG
ROI without location with 80–20 data split (%)							
6 class	BG, N, D, P, S, V	80.01	80.01	82.72	**85.34**	84.81	82.58
5 class	BG, N, D, P, S	81.42	80.71	80.71	81.42	**87.14**	82.28
4 class	BG, N, D, V	**96.89**	93.79	96.12	95.34	96.11	95.65
3 class	D, P, S	**80.00**	71.00	73.00	72.00	78.00	74.80
ROI with location with 80–20 data split (%)							
6 class	BG, N, D, P, S, V	**86.91**	83.24	80.10	83.24	85.34	83.77
5 class	BG, N, D, P, S	80.71	83.57	78.57	82.14	**84.28**	81.85
4 class	BG, N, D, V	94.57	95.34	**96.12**	95.34	**96.12**	95.50
3 class	D, P, S	73.00	78.00	79.00	73.00	**80.00**	76.60
Whole image with 80–20 data split (%)							
4 class	D, P, S, V	77.71	78.37	**79.27**	77.47	78.87	78.34
3 class	D, S, V	90.10	86.81	**94.50**	91.20	86.68	89.86
3 class	D, P, S	79.74	75.94	**81.01**	79.74	74.68	78.22

Significant values are in bold.

In the first scenario, we explored an approach called “ROI without location” with an 80–20 data split. Here, the average accuracy varied across different groupings of classes. The accuracy for a grouping of six classes fluctuated between 80.01 and 85.34%, giving an average of 82.58%. For five classes, it varied from 80.71 to 87.14%, with an average of 82.28%. In a group of four classes, we observed a higher average accuracy of 95.65%, while three classes gave us an average accuracy of 74.80%.

The second method we looked at was “ROI with location”. Here, we noticed a similar pattern to our first method. The six-class grouping showed an average accuracy of 83.77%, with individual tests ranging from 80.10 to 86.91%. The five-class grouping had an average accuracy of 81.85%, ranging between 78.57 and 84.28%. For four classes, the average accuracy was high again at 95.50%, while the three classes gave us an average of 76.60%.

Finally, we examined the “whole image” method with the same 80–20 data split. A four-class grouping resulted in an average accuracy of 78.34%. One group of three classes managed a much higher average accuracy of 89.86%, while the other three-class group had an average accuracy of 78.22%.

Overall, these results show that the different methods and the number of classes used can have varied impacts on performance.

### Robustness and Reliability

To assess the robustness and reliability of our model, we performed multiple tests with varying class distributions on two distinct datasets: the newly created AZH Dataset and the Medetec Dataset. The choice of the Medetec Dataset was influenced by its unique data collection and distribution features, as well as the availability of categories that directly match those in the AZH Dataset, specifically the DPV (Diabetic, Pressure, Venous) categories. This alignment allowed for a consistent evaluation framework, enabling our model to demonstrate its adaptability across similar wound classifications in diverse datasets. These tests were done for Whole image dataset only as wound location information was not available for Medetec dataset.

First, we examined our model on the AZH dataset with a class distribution of 60-15-25 for classes D, P, and V. The model showed notable robustness, achieving an accuracy, precision, recall, and F1-score of 82.69%, 82.52%, 82.69%, and 82.30% respectively. Next, we test it on Medetec dataset. The model continued to showcase excellent robustness, registering an accuracy, precision, recall, and F1-score of 87.50%, 87.44%, 87.50%, and 87.43% respectively as shown in Table 7.

We then altered the class distribution to 70-15-15 on the AZH dataset. The model continued to perform robustly, achieving 87.30% accuracy. Later we tested it on the Medetec dataset, the model held its high performance with accuracy, precision, recall, and F1-score of 88.57%, 88.65%, 88.57%, and 88.50% shown in Table 7.

**Figure 9:**
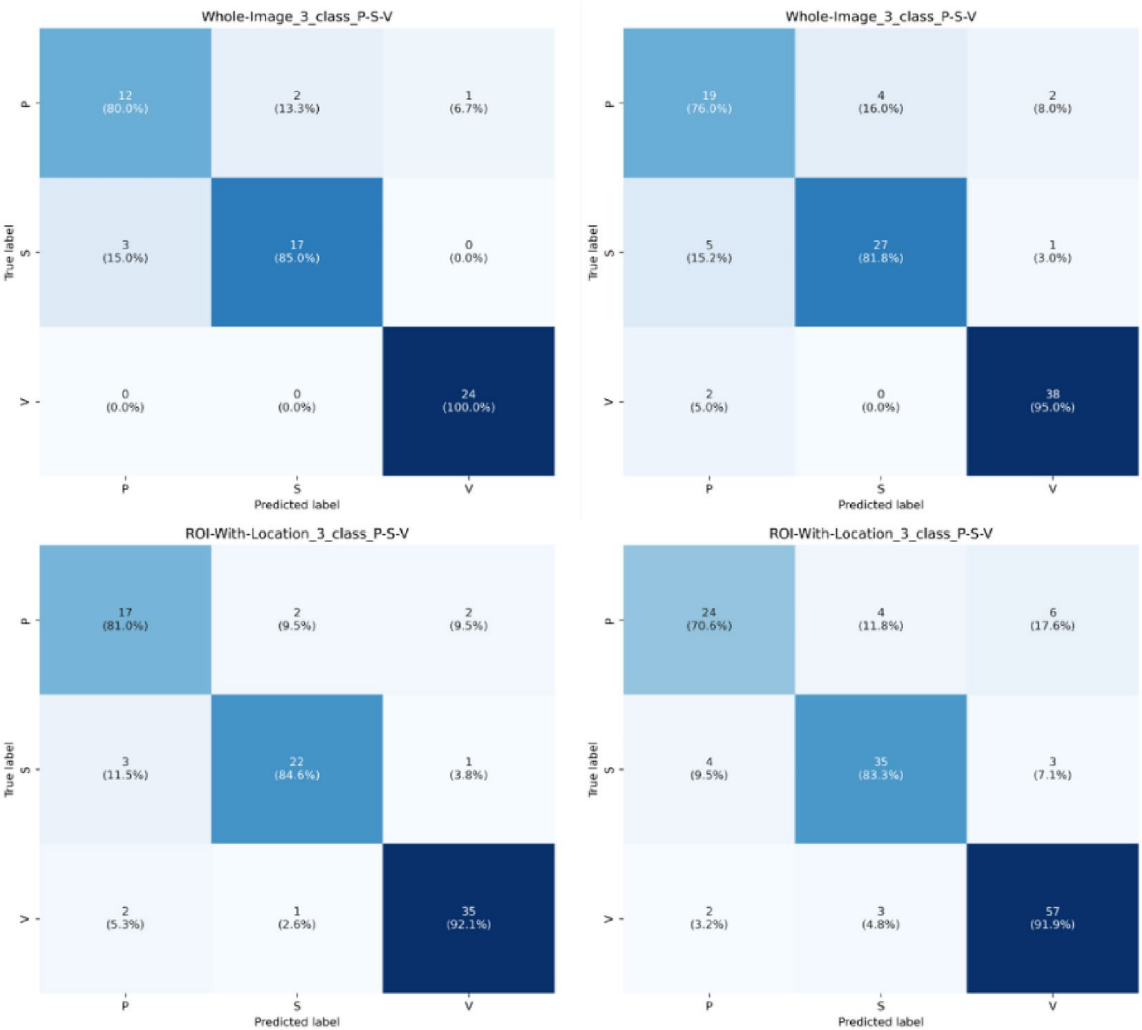
Confusion matrix for three class classification on P-S-V, left column displays dataset with 70/15/15 and right column displays dataset with 60/15/25.

**Figure 10:**
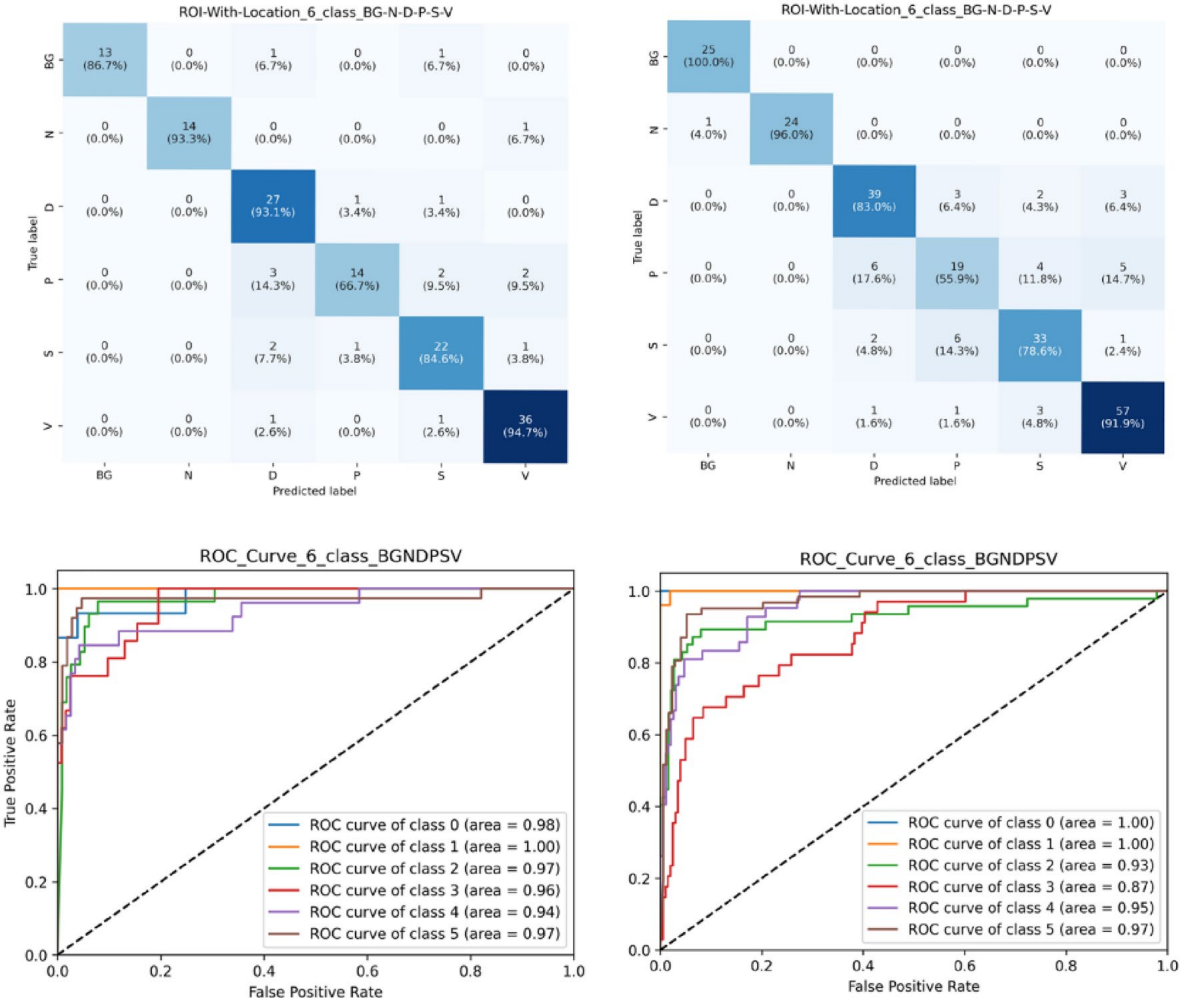
Confusion matrix and ROC curve for six class classification on BG-N-D-P-S-V (class 0–1–2–3–4–5), left column displays dataset with 70/15/15 and right column displays dataset with 60/15/25.

The series of tests reaffirm our model’s consistency and adaptability, demonstrating its ability to perform at a high level regardless of class distribution changes or dataset characteristics. This confirms its robustness and versatility as a data analysis tool (Figs. 9, 10).

## Discussion

### Interpretability

**Figure 11:**
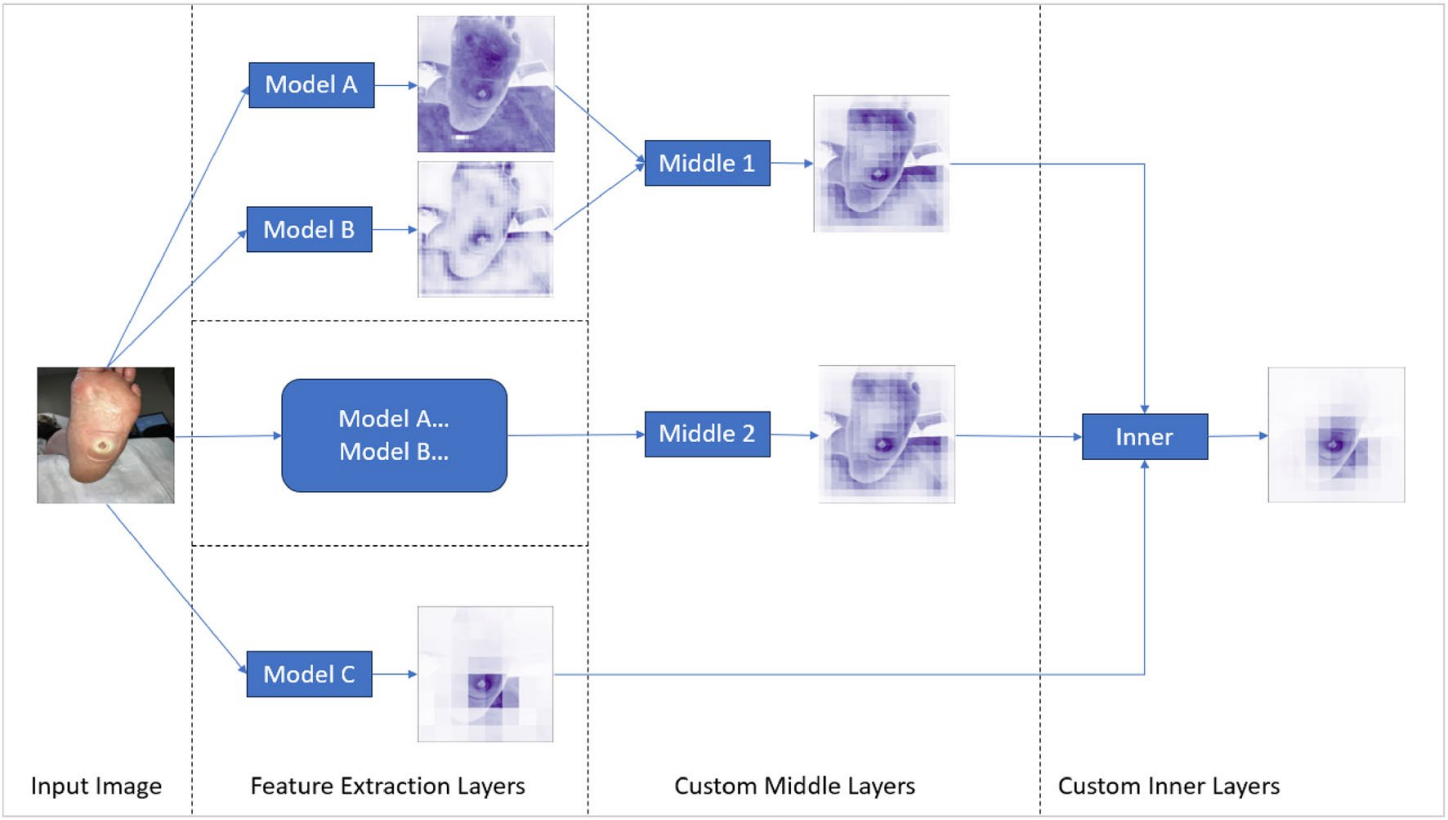
Feature map visualization using LayerGradCAM for few convolutional layers.

Figure 11 provides insight into the interpretability of the model at a granular level, showcasing the last convolutional layers from each feature extraction network—ResNet152, VGG16, and EfficientNet_b2. By employing LayerGradCAM^56^, we can visualize the regions within the image that are most influential for the model’s predictions right before the feature maps are passed to the subsequent stages. This technique highlights the specific activation patterns of the final convolutional layers, offering a focused view on how each network contributes to the decision-making process. These visualizations not only affirm the model’s interpretability but also validate the integrity of the features extracted, ensuring that the most critical aspects of the image data are carried forward to the middle and inner layers for classification.

### Comparison with previous work

Our study presents a comprehensive comparison of our model’s performance with those of previous studies, namely the research conducted by Rostami et al.^36^, Anisuzzaman et al.^28^, Goyal et al.^31^, and Nilsson et al.^32^. The comparison is based on accuracy as the evaluation metric, which is a common criterion for classification tasks. For each work, we have tested our model on the same dataset and compared the results as displayed in Table 9. Figures 9, 10 display confusion matrix for 3-class (P, S and V) and 6-class (BG, N, D, P, S, V). Figure 10 also display ROC plots for 6-class ROI image with location-based image classification.

**Table 9:** Previous work comparison.

Work	No. of classes	Classification	Evaluation metrics	Previous work		Present work	
				Dataset and split size	Result (%)	Dataset and split size	Result (%)
Rostami et al.^36^	6 class	BG, N, D, P, S, V	Accuracy	AZH dataset ROI (NL) (60–15-25)	68.69	AZH Dataset ROI (NL) (60–15-25)	**80.42**
	5 class	BG, N, D, P, V			79.76		**83.41**
		BG, N, D, S, V			84.94		**91.04**
		BG, N, D, P, S			81.49		**84.39**
		BG, N, P, S, V			83.53		**87.23**
	4 class	BG, N, D, V			89.41		**96.22**
		BG, N, P, V			86.57		**91.09**
		BG, N, S, V			**92.2**		91.55
		BG, N, D, P			80.29		**87.02**
		BG, N, D, S			**90.98**		89.2
		BG, N, P, S			84.12		**85.71**
Anisuzzaman et al.^28^	6 class	BG, N, D, P, S, V		AZH dataset ROI (L) (60–15-25) (selected accuracy based on author’s highlight across different models)	82.48	AZH Dataset ROI (L) (60–15-25) Our model is fixed, and we did not used any different combinations	**83.82**
	5 class	BG, N, D, P, V			86.46		**89.11**
		BG, N, D, S, V			91		**91.54**
		BG, N, D, P, S			83.14		**84.39**
		BG, N, P, S, V			86.17		**88.82**
	4 class	BG, N, D, V			95.57		**96.22**
		BG, N, P, V			92.47		**93.15**
		BG, N, S, V			94.16		**96.1**
		BG, N, D, P			89.23		**89.31**
		BG, N, D, S			91.3		**93.52**
		BG, N, P, S			85.71		**88.88**
	3 class	D, S, V			**92**		90.72
		P, S, V			**85.51**		84.05
		D, P, S			72.95		**73.98**
		D, P, V			84.51		**86.71**
	2 class	N, D			**100**		**100**
		N, P			**98.31**		96.61
		N, S			**98.51**		98.5
		N, V			**100**		98.85
		D, P			85		**86.41**
		D, S			89.77		**89.88**
		D, V			94.44		**97.24**
		P, S			**89.47**		84.21
		P, V			90.63		**92.7**
		S, V			97.12		**94.23**
Goyal et al.^31^	2 class	N, D		DFU dataset	92.5	AZH dataset ROI (L)	**100**
						AZH dataset ROI (NL)	**100**
						AZH dataset whole image	**100**
Nilsson et al.^32^	2 class	N, V		A dataset of 300 wound images	85	AZH dataset ROI (L)	**98.85**
		D, V					**97.24**
		P, V					**92.7**
		S, V					**94.23**
						AZH dataset ROI (NL)	**100**
							**97.24**
							**90.62**
							**93.26**
						AZH dataset whole image	**100**
							**96.2**
							**89.23**
							**95.89**

*L* with location, *NL* without location. Significant values are in bold.

In the case of Rostami et al. work^36^, the classification was carried out on a 6-class, 5-class, and 4-class basis using the AZH dataset. Our model outperformed the previous work by a notable margin across all class divisions. For example, in the 6-class division (BG, N, D, P, S, V), our model improved the accuracy by approximately 11.73%. Furthermore, for the 5-class and 4-class divisions, our model consistently showed improvements, highlighting its efficiency and robustness.

Anisuzzaman et al. work^28^ also used the AZH dataset, with a focus on 6-class, 5-class, and 4-class divisions. Our model yielded better accuracy results, such as an increase of about 1.34% in the 6-class division. The consistency of improved performance in all divisions showcases the broad applicability of our model.

As for the work of Goyal et al.^31^, they only classified into a 2-class division (N, D) using the DFU dataset. When tested on the AZH dataset, our model demonstrated 100% accuracy, similar to their findings. This highlights the versatility of our model in achieving high accuracy across different datasets.

Nilsson et al.^32^ conducted their research on a dataset of 300 wound images with a 2-class division. Their model yielded an 85% accuracy rate, while our model, when tested on the AZH dataset, showed a significant improvement in the accuracy rates, ranging from 92.70 to 100%.

### Limitations and future research

While our research demonstrates the strengths of our model, it is not without limitations. For instance, all comparisons in our current study were conducted using the AZH and Medetec dataset. Although our model performed commendably on this dataset, the results might not be fully generalizable to all datasets. Hence, the applicability of our model to other datasets remains an area for further investigation.

It’s noteworthy that our study does not solely rely on accuracy as the evaluation metric. In an attempt to provide a comprehensive evaluation, we also considered other metrics such as precision, recall, and the F1 score. This thorough approach helps to give a well-rounded understanding of our model’s performance. However, despite its strong performance, there could be scenarios or datasets where the model might not yield the same level of success, a potential caveat to be explored in future work.

Future research should be focused on testing the model with larger and more diverse datasets to ensure its generalizability. Specifically, addressing the issue of overlap between healthy and diseased skin, possibly through refining the image preprocessing or feature extraction stages, could yield significant improvements. Furthermore, conducting comparative studies using a wider range of evaluation metrics could offer a broader understanding of the model’s strengths and weaknesses.

In addition to further empirical evaluation, there is also potential to investigate the theoretical properties of the model. Understanding why the model performs as it does could lead to insights that drive further improvements.

### Clinical relevance

Our study, which includes contributions from Jeffrey Niezgoda and Sandeep Gopalakrishnan of Auxillium Health (https://www.auxilliumhealth.ai/), aligns with the innovative approaches of Auxillium Health in leveraging Artificial Intelligence for wound care, demonstrating the practical utility of our multi-modal network in clinical settings. Similar to Auxillium Health’s solutions, which utilize deep learning models for real-time wound monitoring and analytics, our network offers a significant advancement in wound image classification, supporting healthcare providers with reliable, data-driven insights for treatment planning. The incorporation of such AI-based tools in clinical practice, as evidenced by previous authors and applications like those developed by Auxillium Health, underscores the transformative potential of AI in enhancing patient care and outcomes.

## Conclusion

In this study, we presented a multi-modal wound classification network that uniquely incorporates both images and corresponding wound locations to categorize wounds. Differing from previous research, our approach utilizes a pre-existing body map and two datasets to classify wounds based on their locations. Our model is built on a novel deep learning architecture, featuring parallel squeeze-and-excitation blocks (P_scSE), adaptive gated multi-layer perceptron (MLP), axial attention mechanism, and convolutional layers. The integration of image and location data contributed to superior classification outcomes, demonstrating the potential of multi-modal data utilization in wound management. Despite the benefits, our work has some limitations, including data scarcity which affects the generality of our model.

Looking ahead, future research will aim to enhance our model by incorporating more modalities such as pain level, palpation findings, general observations, wound area and volume, and patient demographics. Addressing data overlaps in wound location will also be a priority to enhance classification accuracy. Our efficient wound care algorithm has significant potential for automation in wound healing systems, offering cost-effectiveness and aiding clinicians in prompt diagnosis and development of suitable treatment plans. Especially in resource-scarce areas, AI-enabled wound analysis can contribute to rapid diagnosis and quality treatment. However, this necessitates proper technical training for both patients and physicians, which will also be a focus of future work. Expanding our dataset will help improve our model’s performance and better serve wound care providers and patients alike.

## Data Availability

The AZH dataset can be accessed via the following link: https://github.com/uwm-bigdata/Multi-modal-wound-classification-using-images-and-locations. The Medetec dataset can be accessed via the following link: https://www.medetec.co.uk/files/medetec-image-databases.html?.
